# The impairment of methylmenaquinol:fumarate reductase affects hydrogen peroxide susceptibility and accumulation in *Campylobacter jejuni*

**DOI:** 10.1002/mbo3.158

**Published:** 2014-02-07

**Authors:** Issmat I Kassem, Mahesh Khatri, Yasser M Sanad, Melinda Wolboldt, Yehia M Saif, Jonathan W Olson, Gireesh Rajashekara

**Affiliations:** 1 Food Animal Health Research Program, Ohio Agricultural Research and Development Center, Department of Veterinary Preventive Medicine, The Ohio State UniversityWooster, Ohio; 2 Department of Microbiology, North Carolina State UniversityRaleigh, North Carolina

**Keywords:** *C. jejuni*, catalase, hydrogen peroxide resistance, iron, macrophage, methylmenaquinol:fumarate reductase, Mfr

## Abstract

The methylmenaquinol:fumarate reductase (Mfr) of *Campylobacter jejuni* is a periplasmic respiratory (redox) protein that contributes to the metabolism of fumarate and displays homology to succinate dehydrogenase (Sdh). Since chemically oxidized redox-enzymes, including fumarate reductase and Sdh, contribute to the generation of oxidative stress in *Escherichia coli*, we assessed the role of Mfr in *C. jejuni* after exposure to hydrogen peroxide (H_2_O_2_). Our results show that a Mfr mutant (Δ*mfrA*) strain was less susceptible to H_2_O_2_ as compared to the wildtype (WT). Furthermore, the H_2_O_2_ concentration in the Δ*mfrA* cultures was significantly higher than that of WT after exposure to the oxidant. In the presence of H_2_O_2_, catalase (KatA) activity and *katA* expression were significantly lower in the Δ*mfrA* strain as compared to the WT. Exposure to H_2_O_2_ resulted in a significant decrease in total intracellular iron in the Δ*mfrA* strain as compared to WT, while the addition of iron to the growth medium mitigated H_2_O_2_ susceptibility and accumulation in the mutant. The Δ*mfrA* strain was significantly more persistent in RAW macrophages as compared to the WT. Scanning electron microscopy showed that infection with the Δ*mfrA* strain caused prolonged changes to the macrophages’ morphology, mainly resulting in spherical-shaped cells replete with budding structures and craters. Collectively, our results suggest a role for Mfr in maintaining iron homeostasis in H_2_O_2_ stressed *C. jejuni*, probably via affecting the concentrations of intracellular iron.

## Introduction

*Campylobacter jejuni*, a Gram-negative bacterium, is capable of causing foodborne gastroenteritis and, occasionally, serious neuropathies and other anomalies in humans (Allos 2001). *Campylobacter jejuni* possesses a highly branched electron transport chain (ETC.), which allows the pathogen to survive and adapt to different environmental conditions (Myers and Kelly 2004; Weingarten et al. 2008; Kassem et al. 2012). Individual respiratory proteins which comprise the building blocks of ETC. have been shown to contribute to *C. jejuni*'s metabolism and ability to survive and colonize hosts (Weingarten et al. 2008; Guccione et al. 2010; Kassem et al. 2012). This suggested that the functional attributes of respiratory proteins in *C. jejuni* might be multifaceted and, as such, not fully characterized.

The autoxidation of respiratory proteins in *Escherichia coli* has been implicated in the generation of reactive oxygen species (ROS), including up to 87% of the total H_2_O_2_ produced (Gonzalez-Flecha and Demple 1995; Messner and Imlay 2002). Furthermore, it was specifically shown that the chemical oxidation of flavin adenine dinucleotide (FAD) moieties and the Fe-S clusters of *E. coli*'s fumarate reductase (Frd) and succinate dehydrogenase (Sdh) results in the generation of superoxides and H_2_O_2_, subsequently contributing to oxidative stress (Messner and Imlay 2002). This association between respiratory proteins and oxidative stress in bacteria is not surprising and was further confirmed in a recent study which showed that the deletion of fumarate reductase increased *Bacteroides fragilis*'s aerotolerance (Meehan and Malamy 2012). Of interest is *C. jejuni*'s methylmenaquinol:fumarate reductase (Mfr), a three-subunit periplasmic protein complex (MfrABE) that is transported via the twin arginine translocation (Tat) system (Hitchcock et al. 2010). Notably, Mfr (encoded by *Cj0437*-*0439* in *C. jejuni* NCTC-11168) is not the major fumarate reductase, a function primarily carried out by the FrdCAB complex, in *C. jejuni* and only appears to contribute to a more efficient fumarate-dependent growth (Guccione et al. 2010). Since Mfr is oxygen regulated and exhibits homology to Frd and Sdh, while the MfrA and MfrB subunits possess a FAD moiety and Fe-S centers, respectively (Lemos et al. 2002; Weingarten et al. 2009; Guccione et al. 2010), it is conceivable that the Mfr might also contribute to the generation of oxidative stress in *C. jejuni*. This assumption was supported by our previous study that showed that a Mfr mutant exhibited decreased susceptibility to H_2_O_2_ as compared to the parental strain (Kassem et al. 2012).

Adaptation and resistance to ROS produced in disparate hosts and environments are essential traits for the success of *C. jejuni* as a pathogen (Atack and Kelly 2009). Yet, *C. jejuni* lacks a homologue of OxyR, a common peroxide sensor and a transcriptional regulator of oxidative stress responses in Gram-negative bacteria (Mongkolsuk and Helmann 2002). However, *C. jejuni* possesses PerR, a peroxide stress regulator (van Vliet et al. 1999), and deploys an arsenal of ROS scavengers that primarily include superoxide dismutase (SodB), catalase (KatA), and alkyl hydroperoxide reductase (AhpC) (Atack and Kelly 2009). Additionally, iron metabolism in *C. jejuni* plays an essential part in the oxidative stress response as PerR and other cognate genes were found to respond to iron levels, which also fuel the Fenton reaction and the subsequent formation of hydroxyl radicals (van Vliet et al. 1999; Kim et al. 2011). This is notable because the *mfr* was highly induced under oxygen-limited conditions and the genes encoding Mfr subunits were upregulated in response to exposure to high levels of iron (Stintzi 2003; Palyada et al. 2004; Reid et al. 2008a, b). Therefore, the aforementioned observations raise important questions in regards to the role of Mfr during exposure to H_2_O_2_.

Here, we analyzed the impact of Mfr impairment on H_2_O_2_ stressed *C. jejuni*. For this purpose, we used a strain with a mutation that targeted the gene encoding the MfrA subunit. It was previously shown that the MfrA contains the Tat-signal peptide that is needed to transport the folded Mfr to the periplasm and its impairment results in the loss of Mfr activity (Guccione et al. 2010; Hitchcock et al. 2010). We show that exposure to H_2_O_2_ resulted in (1) decreased catalase activity, (2) decreased total intracellular iron concentrations, and (3) increased accumulation of H_2_O_2_ in the Δ*mfrA* cultures. Collectively, our data suggest that Mfr might indirectly contribute to H_2_O_2_ susceptibility in *C. jejuni*, likely via impacting intracellular iron concentrations. Therefore, our observations expand on Mfr's secondary role in fumarate metabolism and suggest that Mfr might play important additional roles in *C. jejuni*'s pathobiology.

## Materials and Methods

### Bacterial strains and growth conditions

Bacterial strains used in this study are listed in Table 1. *Campylobacter jejuni* strains were cultured on Mueller-Hinton (MH) agar under microaerobic conditions (85% N_2_, 10% CO_2_, 5% O_2_) at 42°C. Incubation at 37°C was performed for assays that included comparison between temperatures. Oxygen-limited/anaerobic conditions were achieved using the BD GasPak Sachets system (BD diagnostics, Franklin Lakes, NJ) as described previously (Kassem et al. 2012). Different oxygen conditions and/or temperatures were used in some assays to be inclusive of varying conditions encountered by *C. jejuni* in disparate hosts and niches (Kassem et al. 2012). Laked horse blood (5%, Oxoid, Lenexa, KS), antibiotics (chloramphenicol: 20 *μ*g mL^−1^, kanamycin: 50 *μ*g mL^−1^), and the *Campylobacter* selective supplement (SR155E, Oxoid) were added to the MH medium when necessary.

**Table 1:** Bacterial strains, plasmids, and primers used in this study. Restriction enzymes and restriction sites in the primer sequences are underlined.

Strain, plasmid, or primer	Properties/Sequence	Source or reference
Strains		
*Campylobacter jejuni* NCTC-11168	Wild-type strain	Dr. Q. Zhang
Δ*mfrA*	Chloramphenicol resistance gene inserted in the *mfrA* (cat^r^)	Weingarten et al. (2009)
C-Δ*mfrA*	Complementation strain. Kanamycin resistant (Kan^r^) and cat^r^	This study
*Escherichia coli* DH5α	Library efficiency competent cells for cloning	Invitrogen
Plasmids		
pIK01	pRY108+*mfrA* used for constructing C-Δ*mfrA*	This study
pRY108	Vector used in complementation studies	Dr. Q. Zhang
Primers		
C-*mfrA*-f	ACGAGGATCCAATGCAATTTATGAATGGAG (*Bam*HI)	This study
C-*mfrA*-r	GTCAGGTACCACAAAT TGCAGATTGACAAG (*Kpn*I)	
*mfrA*-RT-f	GGGCATTTAGCAGGCATTG	Guccione et al. (2010)
*mfrA*-RT-r	GACCATTTCCGCCATTATTTG	
*katA*-RT-f	CAGTAGCAGGTGAAGCAGGTG	IDT^1^
*katA*-RT-r	GCGGATGAAGAATGTCGGAGTG	
*perR*-RT-f	GCTACTCCGCAAAGACTATGTG	IDT^1^
*perR*-RT-r	AGACAGATGATTGACGAGATTG	
*fur*-RT-f	CCATTTCTTTTGGTTCAGCA	IDT^1^
*fur*-RT-r	GCAATCAAGGCTTGCTGTCT	
*rpoA*-RT-f	ATTACAACATCTGCTTATACG	IDT^1^
*rpoA*-RT-r	TCTACTATTTCTTTATTTGATTCG	

1 Integrated DNA Technologies, Inc.

### Construction of a Δ*mfrA* complementation strain

To construct a complementation strain, *mfrA* along with the potential promoter sequence was amplified from the genomic DNA of *C. jejuni* NCTC-11168 using specific primers (Table 1). The primers were designed to include restriction sites that facilitate directional cloning. The PCR products were digested, purified and ligated into a similarly digested pRY108 plasmid using the Fast-Link DNA ligation kit (Epicentre, Madison, WI). The ligated product was then transformed into *E*. *coli* DH5α (Invitrogen, Carlsbad, CA). The resulting plasmid (pIK01) was then purified and introduced into the Δ*mfrA* strain by electroporation as described previously (Wilson et al. 2003). Electroporated cells were spread on MH agar plates supplemented with kanamycin and chloramphenicol and incubated at 42°C for 3 days under microaerobic conditions. The resulting colonies were harvested and streak purified, and one colony (C-Δ*mfrA*), which was PCR-positive for the presence of *mfrA*, was selected for further studies.

### Susceptibility to oxidative and nitrosative stresses

The susceptibility of the *ΔmfrA* strain to oxidative stress was determined using a diffusion assay (Atack et al. 2008). A 100 *μ*L of each of the *C. jejuni* cultures (OD_600_ of 1.0) were spread onto MH agar plates. A hole (5 mm in diameter) was aseptically created in the center of the plates and filled with 30 *μ*L of 3% H_2_O_2_ (Rajashekara et al. 2009). The plates were then incubated at 37 or 42*°*C under microaerobic or anaerobic conditions. The diameter of the zone of inhibited growth was measured after 48 h of incubation. Similarly, the *ΔmfrA* strain was tested for susceptibility to other oxidative stressors, including paraquat (10 mmol/L), *tert*-butylhydroperoxide (100 mmol/L), and cumene hydroperoxide (10%) (Sigma-Aldrich, St. Louis, MO) (Atack et al. 2008; Rajashekara et al. 2009).

To investigate if iron played a role in H_2_O_2_ susceptibility, the *ΔmfrA* strain was challenged with H_2_O_2_ in a medium containing relatively high iron concentrations as described elsewhere (Ishikawa et al. 2003). For this purpose, 40 *μ*mol/L (final concentration) of ferrous sulfate (Sigma-Aldrich) was added to the MH agar and the susceptibility to H_2_O_2_ was assessed as described above.

To test if the deletion of *mfrA* impacted *C. jejuni*'s ability to tolerate nitrosative stress, cultures suspended in MH broth (OD_600_ of 0.3) were challenged with 10 mmol/L sodium nitroprusside (Atack et al. 2008) for 30 min at 42°C under microaerobic conditions. The cultures were then serially diluted (10-fold) in MH broth and 100 *μ*L of each dilution was spread onto MH agar plates, incubated for 48 h under microaerobic conditions, and the number of colony-forming units (CFU) was calculated.

All experiments were repeated at least three times and samples were tested in triplicate in each experiment.

### Aerotolerance assay

The tolerance of the *ΔmfrA* strain to aerobic (ambient oxygen) conditions was assessed as described previously (Atack et al. 2008; Fields and Thompson 2008). Briefly, *C. jejuni* cultures were adjusted to OD_600_ of 0.1 in MH broth and incubated shaking (200 rpm) in an aerobic atmosphere at different temperatures (25*°*C, 37*°*C, and 42*°*C, respectively). Measurements of OD_600_ were recorded after 24, and 48 and 72 h of incubation (data not shown). Since the aerotolerance phenotype was most pronounced at 37*°*C, we quantified the surviving CFU at this temperature using serial dilution as described above. The experiments were repeated at least three times and samples were tested in triplicate in each experiment.

### Catalase activity

Quantification of catalase activity in *C. jejuni* cultures before and after challenge with H_2_O_2_ was performed using the OxiSelect™ catalase activity assay kit (Cell Biolabs, Inc., San Diego, CA). This kit allows the colorimetric detection of H_2_O_2_ through a two step process, which includes the generation of a quinoneimine dye that can be measured using a spectrophotometer (*λ* = 550). Briefly, *C. jejuni* cultures were grown to an early stationary phase and each culture was divided into two equal volumes (10 mL each) and grown for a further 15 min either in the absence or presence of 2 mmol/L H_2_O_2_. The cultures were concentrated to an OD_600_ of 1.5 and 1 mL of each culture was then centrifuged, the pellets were lysed using the Peripreps™ Periplasting kit (Epicentre) to prepare crude cell extracts as described elsewhere (Flint et al. 2012). The crude lysates (∼100 ng *μ*L^−1^) were then assayed for catalase activity, and the catalase concentrations were determined using a standard curve as described in the manual of the OxiSelect™ catalase activity assay kit. All samples were tested in triplicate and the experiment was repeated three times.

### Accumulation of H_2_O_2_ in the *C. jejuni* liquid cultures

The accumulation of H_2_O_2_ in *C. jejuni* broth cultures was assessed using the FOX reagent as described previously (Hayashi et al. 2011). The FOX reagent is composed of 90% sorbitol, 25 mmol/L H_2_SO_4_, 250 mmol/L ferrous sulfate, and 100 mmol/L xylene orange and allows a sensitive colorimetric detection of peroxides, which can be quantified using a spectrophotometer (*λ* = 560 nm) (Wolff 1994; Hayashi et al. 2011). Briefly, *C. jejuni* cultures were grown to an early stationary phase and adjusted to OD_600_ of 0.4. Each culture was then divided into two equal volumes (5 mL each) and incubated for a further 1 h either in the absence (control) or presence of H_2_O_2_ (2 mmol/L final concentration) as a stressor. A volume of 100 µL of each culture was added to 900 *μ*L of the FOX reagent, mixed thoroughly, and incubated for 30 min at room temperature. The mixture was then transferred to plastic cuvettes and the OD_560_ of each sample was determined using a spectrophotometer. Cuvettes that contained 100 *μ*L of sterile MH broth and 900 *μ*L of the FOX reagent were used as blank. A standard curve was used to convert OD measurements to H_2_O_2_ concentrations.

The dose-dependent impact of iron on H_2_O_2_ accumulation in the cultures of the Δ*mfrA* strain was also assessed using the FOX reagent. For this purpose, cultures of the Δ*mfrA* strain were incubated with or without iron, using two different iron doses (40 *μ*mol/L and 80 *μ*mol/L), and H_2_O_2_ for 45 min (Ishikawa et al. 2003). A similar setup included the addition of the iron chelator deferoxamine mesylate (Desferal; 20 *μ*mol/L final concentration) (Ishikawa et al. 2003) instead of iron. H_2_O_2_ accumulation was then measured as described above using appropriate blanks (containing MH with iron or Desferal) for each setup. The experiments were repeated at least three times and samples were tested in triplicate in each experiment.

### Measurement of total intracellular iron concentration

The concentration of total intracellular iron in the *C. jejuni* cultures was measured using the ThermoFinnigan Element 2 inductively coupled plasma sector field mass spectrometer (Saito and Schneider 2006) at the Trace Element Research Laboratory (TERL, the Ohio State University, OH; http://www.geology.ohio-state.edu/marc/index.html). Briefly, *C. jejuni* cultures were adjusted to OD_600_ of 0.4 in 150 mL of MH broth. The cultures were then challenged with H_2_O_2_ as described above for 45 min in microaerobic conditions at 42°C. The cultures were centrifuged at 4000*g* for 10 min and the pellets were washed three times with sterile Milli-Q water (EMD Millipore Corporation, Billerica, MA). The pellets were then immediately frozen at −80°C and transferred to TERL for further analysis. Pellets were then transferred to a 15 mL polypropylene tube (with screw cap) and digested in 1 mL concentrated ultrapure nitric acid in a boiling water bath for 30 min. This was followed by diluting the digested samples to 100 mL total volume with deionized water. Before measurement, 10 ppb indium was added as an internal control to all samples. Samples were analyzed in triplicate, while the experiment was repeated twice.

### qRT-PCR analysis

The expression of *katA* (*Cj1385*), *fur* (the ferric uptake regulator gene; *Cj0400*) and *perR* (*Cj0322*) was investigated in the wildtype and the *ΔmfrA* strain before and after exposure to H_2_O_2_ and iron. For this purpose, *C. jejuni* cultures were incubated with and without added iron and/or H_2_O_2_ for 15 min (Palyada et al. 2009) with shaking (200 rpm) at 42°C. The cells were then pelleted by centrifugation and RNA was extracted using RNeasy Mini Kit (Qiagen, Valencia, CA). cDNA was synthesized using SuperScript® III First-Strand Synthesis SuperMix (Invitrogen). Finally, qPCR analysis was performed using SensiMixPlus® SYBR RT-PCR Kit (Quantace, Los Angeles, CA). The relative levels of gene expression were normalized with those of *rpoA* (internal control) and the comparative threshold cycle (CT) method was used to report the difference in transcript levels.

### Infection of macrophages

Because the mouse leukaemic monocyte macrophages (RAW 264.7; ATCC # TIB-71™) generate a respiratory burst in the presence of *C. jejuni* (Day et al. 2000), we assessed the impact of *mfrA* deletion on *C. jejuni*'s interaction with these cells. Survival of the *C. jejuni* strains in macrophages was performed as described elsewhere (Lin et al. 2009). Briefly, *C. jejuni* cultures were added to macrophage monolayers to achieve a multiplicity of infection (MOI) of 100 in 24-well tissue culture plates, which were incubated at 37 ºC with 5% CO_2_. After 3 h incubation, the infected monolayers were washed three times with minimum essential medium (MEM) supplemented with 1% FBS and covered with 1 mL of the same medium supplemented with gentamicin (150 *μ*g mL^−1^) for 1 h. The monolayers were then washed three times and covered with the aforementioned medium supplemented with gentamicin (10 *μ*g mL^−1^). At 1, 4, and 8 h post infection, the cells were lysed using 0.1% (v/v) Triton X-100, serially diluted (10-folds), and 100 *μ*L of each dilution was spread on MH agar plates. The plates were then incubated for 48 h at 42°C under microaerobic conditions. The number of surviving bacteria was determined by counting CFUs.

Since nitric oxide (NO) is produced by stimulated macrophages, the release of NO from macrophages was measured at eight hours post infection with the *C. jejuni* strains using the Griess reagent kit (Promega, Madison, WI) as described previously (Sun et al. 2003; Khatri et al. 2009). All macrophage-related experiments were repeated three times and each strain was tested in duplicate per assay.

### Scanning electron microscopy

To further investigate the interaction between the Δ*mfrA* strain and the RAW macrophages, infected monolayers were analyzed using scanning electron microscopy (SEM) as described previously (Van Deun et al. 2008) with minor modifications. Briefly, the macrophages were grown on HCl treated glass coverslips. The *C. jejuni* strains were added to the monolayers at an MOI of 200. After 3 h of incubation, the cells were gently washed with 1× PBS and fixed (3% glutaraldehyde, 2% paraformaldehyde in 0.1 mol/L potassium phosphate buffer, pH 7.2) at 4°C overnight. The samples were then rinsed in 0.1 mol/L potassium phosphate (three times with 15 min incubation for each step) and postfixed with 1% osmium tetroxide for 1 h at room temperature in the dark. This was followed with serial dehydration of the samples in ethanol, critical point drying and platinum sputter-coating (Molecular and Cellular Imaging Center, Ohio Agricultural Research and Development Center [OARDC]; http://www.oardc.ohio-state.edu/mcic). The samples were visualized and imaged using the Hitachi S-4700 scanning electron microscope (Tokyo, Japan). All samples were tested in duplicate and noninfected monolayers were used as controls to assess morphological changes associated with the bacterial infection.

### Infection of chickens

The impact of *mfrA* deletion on *C. jejuni*'s capability to infect chickens was assessed (Hendrixson and DiRita 2004). One-day-old chickens (specific pathogen free; SPF) were confirmed *Campylobacter*-free by testing cloacal samples prior to infection. The chickens were divided into four groups, each containing seven birds (*n* = 28). To test a possible impact associated with the dose of the bacterial inoculum, chickens were inoculated orally either with ∼2 × 10^3^ (low dose, LD) or ∼2 × 10^7^ (high dose, HD) CFU of each strain tested. Seven days post inoculation, the chickens were euthanized and the ceca were aseptically collected, weighed, and homogenized in 1× PBS. The cecal extracts were serially diluted (10-fold) and 100 *μ*L from each dilution were spread onto MH agar plates supplemented with the *Campylobacter* selective supplement; SR117E (Oxoid). The plates were then incubated at 42°C under microaerobic conditions for 48 h and the number of CFU g^−1^ of cecal content was calculated. The chickens were cared for according to the guidelines of the Association for the Assessment and Accreditation of Laboratory Animal Care (AAALAC).

### Statistics

Data were expressed as mean ± standard deviation and statistical analysis was performed using a one-way analysis of variance followed by Tukey's posttest. In cases where only two sets of data were compared the Student's *t*-test was used for analysis, while the Mann–Whitney rank sum test was used to compare groups in the chicken colonization experiment. A *P* value of <0.05 was considered statistically significant.

## Results

### The deletion of *mfrA* decreased *C. jejuni*'s susceptibility to H_2_O_2_ and ambient oxygen but not to organic peroxides and paraquat

**Figure 1:**
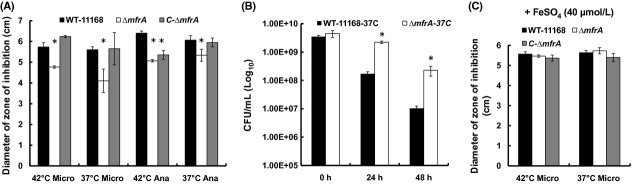
Susceptibility of the *Campylobacter jejuni* strains to H_2_O_2_ and ambient oxygen. (A) Decreased susceptibility of the Δ*mfrA* strain to H_2_O_2_. Microaerobic and anaerobic incubation conditions are abbreviated as “Micro” and “Ana,” respectively. (B) Survival of the Δ*mfrA* strain under ambient oxygen (aerotolerance) at 37°C. (C) Addition of exogenous iron restores the H_2_O_2_ susceptibility of the Δ*mfrA* strain to levels comparable to those of the wildtype. Statistically significant (*P* < 0.05) differences are highlighted with “*”. All assays were repeated three times independently and samples were tested in at least three replicates per experiment. Data are presented as mean ± standard deviation.

To expand our hypothesis that impairment of Mfr might impact *C. jejuni*'s susceptibility to oxidative stressors, the Δ*mfrA* strain was challenged with different oxidants. Our results show that the Δ*mfrA* strain was significantly less susceptible (*P *<* *0.05) to H_2_O_2_ as compared to the wildtype (Fig. 1A). However, the mutant displayed a susceptibility to cumene hydroperoxide, *tert*-butylhydroperoxide, and paraquat that was similar to that of the wild-type strain (see Fig. S1).

Since the deletion of *mfrA* decreased *C. jejuni*'s susceptibility to H_2_O_2_, we assessed whether the survival of the mutant was also affected under ambient oxygen (aerotolerance) (Atack et al. 2008; Fields and Thompson 2008). Our results show that the Δ*mfrA* strain was significantly less susceptible to ambient oxygen concentrations as compared to the wildtype regardless of the incubation temperature (data not shown and Fig. 1B).

Paraquat was used to generate superoxide anions (Bagley et al. 1986), which are primarily scavenged by *C. jejuni*'s SOD (superoxide dismutase) and converted to H_2_O_2_. However, since the deletion of *mfrA* did not affect the susceptibility to paraquat, it can be concluded that the mutation did not directly and/or significantly impact SOD-associated activity. Furthermore, previous studies showed that a *C. jejuni ahpC* mutant was hypersensitive to cumene hydroperoxide and exhibited reduced aerotolerance (Baillon et al. 1999). Since AhpC primarily scavenges organic peroxides but not H_2_O_2_ in *C. jejuni* (Baillon et al. 1999), it can be concluded that the decreased susceptibility of the *ΔmfrA* strain to H_2_O_2_ and ambient oxygen concentration was likely not directly related to AhpC.

### Catalase activity did not increase in the Δ*mfrA* strain after exposure to H_2_O_2_

**Figure 2:**
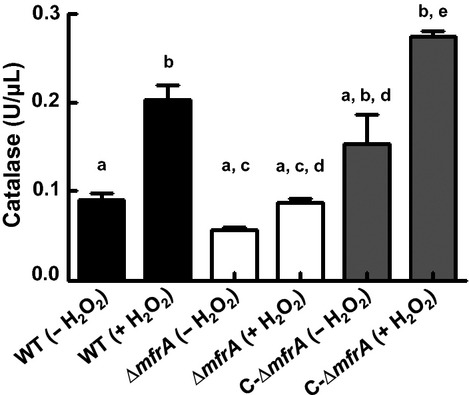
Measurement of the catalase activity in the *Campylobacter jejuni* strains before and after exposure to H_2_O_2_. Catalase activity in the Δ*mfrA* strain did not significantly increase upon exposure to H_2_O_2_. The catalase activity was measured in crude cell extracts using the OxiSelect™ catalase activity assay kit. Different letters indicate statistically significant (*P *<* *0.05) differences. For example, a bar that is highlighted by the letter “a” is significantly different than those highlighted by “b” and “c,” while the same letter indicate comparisons that were not statistically significant. The assay was repeated three times independently and samples were tested in three replicates per experiment. Data are presented as mean ± standard deviation.

*Campylobacter jejuni* possesses a single catalase (KatA) that breaks down H_2_O_2_ into water and oxygen (Atack and Kelly 2009). Therefore, we assessed the activity of the catalase in the mutant strain before and after exposure to H_2_O_2_. Our results show that the catalase activity in the Δ*mfrA* strain was similar to that of the wildtype before exposure to H_2_O_2_ (Fig. 2). However, after the addition of H_2_O_2_, the catalase activity in the mutant was significantly (*P *<* *0.05) lower than that in the wildtype (Fig. 2) and was similar to pre-exposure levels. These results were unexpected, because catalase is normally induced by H_2_O_2_ and superoxide anions (Grant and Park 1995; Garenaux et al. 2008).

### Hydrogen peroxide accumulates in the cultures of the Δ*mfrA* strain

**Figure 3:**
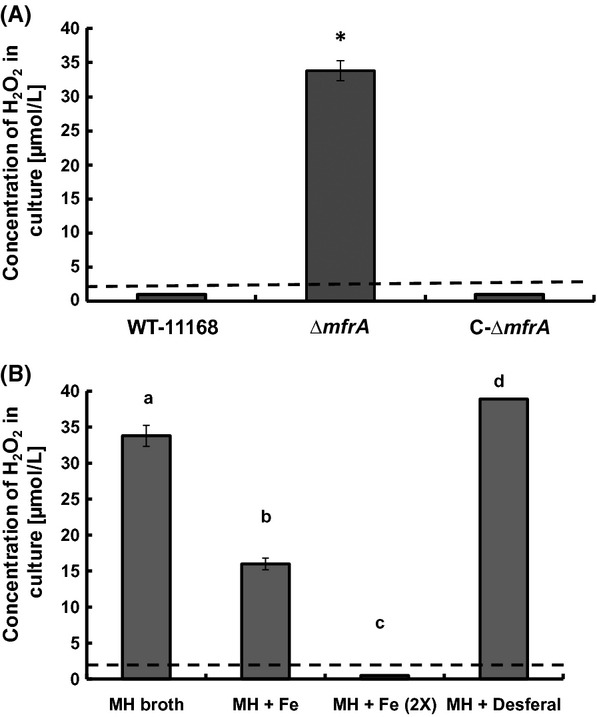
Accumulation of H_2_O_2_ in *Campylobacter jejuni* cultures. (A) Quantification of the concentrations of H_2_O_2_ that accumulated in the *C. jejuni* strains after exposure to the oxidant. (B) Accumulation of H_2_O_2_ in the cultures of the Δ*mfrA* strain after the addition of iron in two doses (MH + Fe and MH + Fe [2×], which correspond to 40 *μ*mol/L and 80 *μ*mol/L iron, respectively) and desferal, respectively. A similar analysis could not be performed on the wildtype, because H_2_O_2_ levels were below the detection limit. Dashed line indicates the detection limit of the assay. Statistically significant (*P *<* *0.05) differences are highlighted by “*”. Assays were repeated three times independently and samples were tested in three replicates per experiment. Data are presented as mean ± standard deviation.

To confirm the aforementioned catalase activity in the Δ*mfrA* strain, the accumulation of H_2_O_2_ was measured in the broth of the growing cultures (Hayashi et al. 2011). Our results show that H_2_O_2_ concentrations in the MH broth culture of the *ΔmfrA* strain were significantly higher (*P *<* *0.05) than those of the wildtype after challenging the strains with a known concentration of the oxidant (Fig. 3). This trend was also observed when the experiment was performed using the MEM alpha (MEMα) instead of MH broth (data not shown). These results indicated defects in H_2_O_2_ breakdown in the *ΔmfrA* strain as compared to the wildtype, which corroborated the lower catalase activity observed in the mutant cells.

### Intracellular iron concentrations in the Δ*mfrA* strain decrease after exposure to H_2_O_2_ and the addition of exogenous iron mitigates the susceptibility of the Δ*mfrA* strain to H_2_O_2_

**Figure 4:**
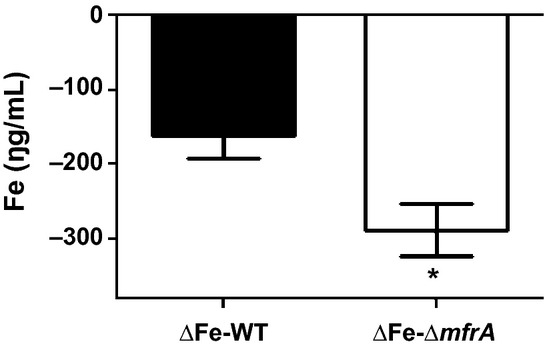
The impact of iron on the interaction of the Δ*mfrA* strain with H_2_O_2_. Quantification of the decrease in total intracellular iron concentrations in the *Campylobacter jejuni* strains after exposure to H_2_O_2_. ΔFe-WT and ΔFe-Δ*mfrA* designate H_2_O_2_-associated drop in the iron concentrations in the wild-type strain and the Δ*mfrA* strain, respectively. Statistically significant (*P *<* *0.05) differences are highlighted by “*”. The assays were repeated twice independently and samples were tested in triplicate per experiment. Data are presented as mean ± standard deviation.

Iron concentrations impact catalase activity, and it was previously reported that the expression of *katA* was repressed by iron (van Vliet et al. 1999; Palyada et al. 2004). Therefore, we investigated if total intracellular iron levels might be impacted by the deletion of *mfrA*. Our results showed a decrease in total intracellular iron concentration in both the wildtype and mutant cells only after exposure to H_2_O_2_ (Fig. 4). This was not surprising as intracellular iron levels were reported to decrease in other bacteria after exposure to oxygen (Yamamoto et al. 2004). However, the decrease in total iron concentration was significantly greater in the mutant cells as compared to those of the wildtype (Fig. 4A).

Supplementing the MH growth medium with iron increased the susceptibility of the *ΔmfrA* strain to H_2_O_2_ to levels comparable to those of the wildtype (Fig. 1C). Furthermore, the addition of iron resulted in a dose-dependent decrease in H_2_O_2_ accumulation in the MH growth medium of the mutant, while exposure to an iron chelator (deferoxamine mesylate) (Ishikawa et al. 2003) resulted in a statistically significant increase in H_2_O_2_ accumulation in the mutant cultures (Fig. 3B). A similar trend was also observed when analysis was conducted using MEMα instead of MH broth (data not shown). Taken together, our data suggest that the deletion of *mfrA* impacted the levels of total intracellular iron in cells exposed to H_2_O_2_.

### qRT-PCR analysis of the expression of selected genes in the *C. jejuni* strains before and after exposure to H_2_O_2_ and iron

**Figure 5:**
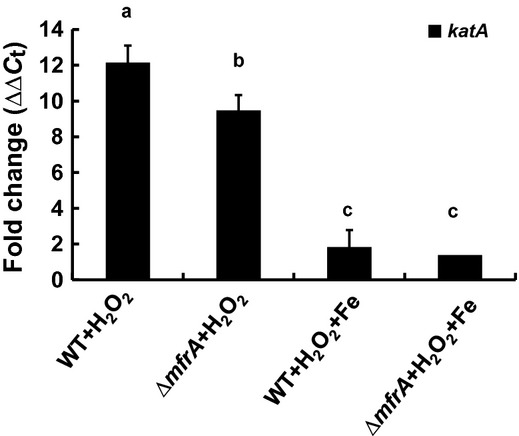
Quantification of the expression of *katA* in *Campylobacter jejuni* before and after exposure to H_2_O_2_ and iron. Expression of *rpoA* was used as a control and the difference in expression was calculated using the ΔΔCt method. The experiment was repeated twice and all samples were tested in triplicate. Statistically significant (*P *<* *0.05) differences are highlighted with different letters. For example, a bar that is highlighted by the letter “a” is significantly different than those highlighted by “b” and “c,” while the same letter indicates comparisons that were not statistically significant. Data are presented as mean ± standard deviation.

Previous work showed that PerR suppresses *katA* in an iron-dependent manner (van Vliet et al. 1999; Kim et al. 2011), while the impairment of *perR* resulted in increasing *C. jejuni*'s resistance to peroxide stress via the derepression of *katA* (van Vliet et al. 1999). Furthermore, it was shown that *katA* was coregulated by *perR* and *fur* (van Vliet et al. 1999; Palyada et al. 2004). Therefore, we quantified the expression of *katA*,*fur*, and *perR* in the wildtype and the Δ*mfrA* strain in the presence and absence of H_2_O_2_ and iron. Our results show that the expression of *katA* was significantly higher (∼2.7-fold; *P *<* *0.05) in the wildtype as compared to the Δ*mfrA* strain after exposure to H_2_O_2_ (Fig. 5). However, there was no significant difference in *katA* expression when the strains were exposed to H_2_O_2_ and iron (Fig. 5). The expression of *fur* and *perR* did not significantly vary between the wildtype and the Δ*mfrA* strain under the experimental conditions used in this study (data not shown). We also investigated the expression of *mfrA* in the wildtype and found that exposure to H_2_O_2_ significantly increased the expression of this gene by approximately twofold (data not shown). The qRT-PCR analysis corroborated our observations that the catalase activity was lower in the Δ*mfrA* strain after H_2_O_2_ exposure and that iron might contribute to the phenotypes of the H_2_O_2_-treated mutant strain.

### The Δ*mfrA* strain persisted in RAW macrophages

**Figure 6:**
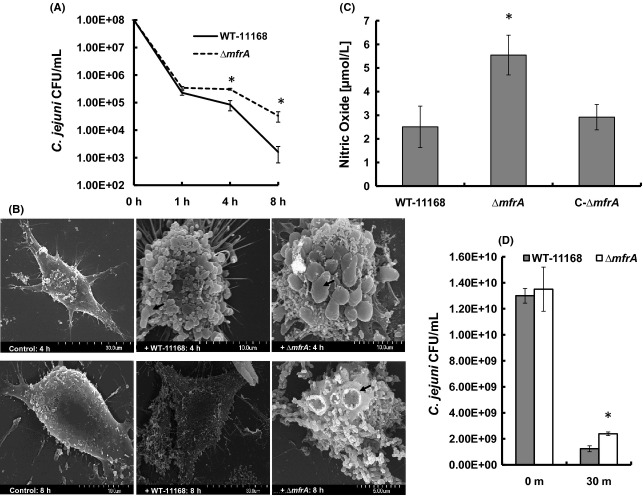
The interaction of *Campylobacter jejuni* strains with murine macrophages (RAW 264.7 cells). (A) Enumeration of *C. jejuni*CFUs that survived inside the macrophages using the gentamicin protection assay. (B) Scanning electron microscopy showing macrophages infected with the *C. jejuni* strains. Monolayers that were not infected are labeled as control. Note the ruffled surface of infected macrophages and the black arrows show budding bodies and craters on the surface of the macrophages. At 8 h, macrophages challenged with the wildtype showed a decrease in ruffled surface and budding structures. (C) The production of nitric oxide in macrophage cultures challenged with the *C. jejuni* strains. (D) Quantification of *C. jejuni*CFUs that survived exposure to sodium nitroprusside (10 mmol/L) for 30 min. Survival in macrophages, NO production, and resistance to nitroprusside assays were repeated three times independently and samples were tested in three replicates per experiment. The SEM assay was repeated twice and the samples were analyzed in duplicate. Statistically significant (*P *<* *0.05) differences are highlighted with “*” and data are presented as mean ± standard deviation.

Macrophages are known to produce respiratory/oxidative bursts in response to pathogens (Santos et al. 1999; Day et al. 2000). Subsequently, the interaction of the Δ*mfrA* strain with murine RAW 264.7 macrophages was assessed as described previously (Day et al. 2000). Our results show that the mutant was significantly (*P *<* *0.05) more persistent in the macrophages at 4 and 8 h post infection as compared to the wildtype (Fig. 6A). The CFU number of the Δ*mfrA* strain retrieved at 8 h post infection was approximately 2 log higher than that of the wildtype (Fig. 6A).

We also investigated whether the persistence of the Δ*mfrA* strain in macrophages might lead to prolonged gross changes to the morphology of the eukaryotic cells. SEM analysis showed that both the wildtype and the Δ*mfrA* strain resulted in a drastic change in the macrophages’ morphology, mainly a formation of spherical-shaped cells with a surface replete with budding (apoptotic) structures and craters, as compared to noninfected cells (Fig. 6B). However, at 8 h post infection, the majority of the macrophages incubated with the wildtype possessed a phenotype similar to the noninfected cells, while the macrophages incubated with the Δ*mfrA* strain still exhibited the budding structures and craters (Fig. 6B). Furthermore, we observed that the production of NO in the mutant strain-macrophage co-cultures was significantly higher (*P *<* *0.05) as compared to those infected with the wildtype at 8 h post infection (Fig. 6C), which probably highlighted a response from the macrophage to the persistence of the mutant in the co-cultures. However, the Δ*mfrA* strain exhibited a slight but statistically significant decrease in susceptibility to sodium nitroprusside (Fig. 6D), a compound that is capable of releasing NO (Grossi and D'Angelo 2005). Therefore, the persistence of the Δ*mfrA* strain in the macrophages might be feasible due to the mutant's decreased susceptibility to the components of the oxidative burst; NO and superoxides that dismutate into H_2_O_2_.

### The Δ*mfrA* strain was not defective in the colonization of chickens

**Figure 7:**
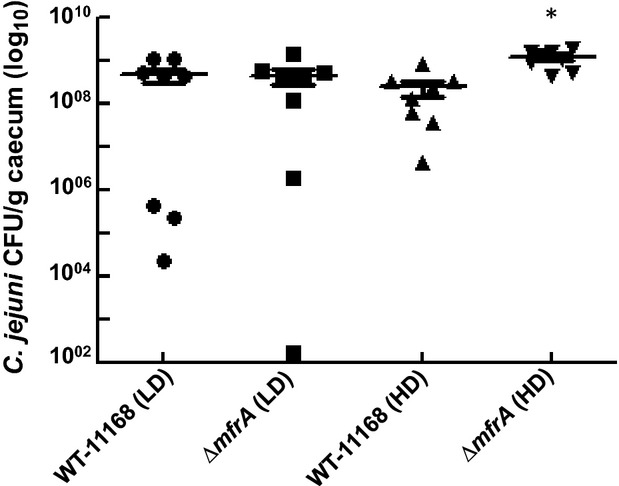
Chicken colonization ability of the Δ*mfrA* strain as compared to WT. HD and LD refer to high inoculum dose (∼2 × 10^7^ CFU) and low inoculum dose (∼2 × 10^3^ CFU), respectively. The ceca of infected chickens were collected 7 days post inoculation and the content was serially diluted and spread onto MH agar plates supplemented with the *Campylobacter* selective supplement. Statistically significant (*P *<* *0.05) differences are highlighted with “*”.

Mfr contributes to the reduction in fermentation byproducts in the host, which suggested that the impairment of Mfr might lead to reduced colonization of chickens (Guccione et al. 2010). However, a previous study reported that the ability of a Δ*mfrA* strain to colonize chickens was similar to that of the wildtype (Weingarten et al. 2009). To reconcile these studies, we hypothesized that there may exist an inoculation dose-dependent impact associated with the Δ*mfrA* strain's ability to colonize the chickens. Therefore, we inoculated the chickens with different doses of the *C. jejuni* strains. Our results showed that at lower doses (∼2 × 10^3^ CFU), the wildtype and the mutant CFU numbers retrieved from the ceca were not statistically different (Fig. 7). At a relatively higher dose (∼2 × 10^7^ CFU), the CFUs of the Δ*mfrA* strain were retrieved in numbers that were significantly higher (*P *<* *0.05) but still comparable to those of the wildtype (Fig. 7).

## Discussion

Since KatA is the major enzyme involved in H_2_O_2_ detoxification in *C. jejuni* (Bingham-Ramos and Hendrixson 2008), we hypothesized that the deletion of *mfrA* might impact catalase expression and/or activity, resulting in the decreased susceptibility of the mutant to H_2_O_2_. This was supported by reports showing that a *perR* mutant was hyper-resistant to H_2_O_2_ via the derepression of *katA* (van Vliet et al. 1999), while a Δ*katA* mutant was extremely (10,000-fold) sensitive to the oxidant (Palyada et al. 2004). Our data showed that both the catalase expression and activity were lower in the Δ*mfrA* strain after exposure to H_2_O_2_ (Figs. 2,5), while H_2_O_2_ accumulated in higher concentrations in the cultures of the Δ*mfrA* strain (Fig. 3A). This suggested that H_2_O_2_ was not being broken down as efficiently as in the wildtype. Therefore, these observations did not conform to our initial expectation that the decreased susceptibility of the Δ*mfrA* strain to H_2_O_2_ resulted from increases in *katA* expression and/or KatA activity. It is important to note that a previous study reported that exposure to H_2_O_2_ and iron resulted in 132-fold increase in *katA* expression in the wildtype strain (Palyada et al. 2009), which was substantially higher than the expression levels observed in our study. However, Palyada et al. (2009) conducted their experiments in iron-restricted MEMα as opposed to the MH broth used in our studies. Since Mueller-Hinton broth constituently contains iron (Ishikawa et al. 2003), this might explain the different expression levels of *katA* reported in this study. Regardless, although the impairment of *mfrA* affected the catalase expression and activity, our findings do not provide direct evidence for the association of catalase with the decreased susceptibility of the Δ*mfrA* strain to H_2_O_2_.

FAD moieties and Fe-S centers of proteins, including periplasmic reductases such as the Mfr, can interact with H_2_O_2_ and contribute to the generation of ROS via the release of iron, which fuels the Fenton reaction and the production of highly toxic hydroxyl radicals (Beinert et al. 1997; Hidalgo et al. 1997; Lemos et al. 2002; Andrews et al. 2003; Kiley and Beinert 2003; Guccione et al. 2010; Stahl et al. 2012). Therefore, an explanation for decreased susceptibility of the Δ*mfrA* strain to H_2_O_2_ would be a possible impairment of the Fenton reaction via a decrease in the availability of total intracellular iron. This may also apply to the aerotolerance phenotype of the Δ*mfrA* strain, because peroxide can be produced endogenously under aerobic conditions (King et al. 2000; Gibson et al. 2000), which would then trigger various responses, including interaction with iron, oxidation of Fe-S clusters, and generation of hydroxyl radicals (King et al. 2000; Gibson et al. 2000). Indeed, the total iron concentration in the Δ*mfrA* mutant cells was lower than that of the wildtype after exposure to H_2_O_2_ (Fig. 4). Additionally, the accumulation of H_2_O_2_ in the mutant decreased, while its susceptibility to the oxidant increased to levels comparable to those of the wildtype in an iron-replete medium (Figs. 1C, 3B). Furthermore, *katA* expression was statistically similar in the wildtype and the Δ*mfrA* strain in the presence of both H_2_O_2_ and high iron concentration (Fig. 5), while the catalase activity showed a similar trend in iron-replete medium (data not shown). Since the deletion of *mfrA* that carries the Tat-signal motif (SRRDFIK) would impair the export of the Mfr complex to the periplasm (Guccione et al. 2010; Hitchcock et al. 2010), the localization and maturation of the Fe-S containing Mfr subunits (MfrB) in the mutant cells will be affected (Guccione et al. 2010; Hitchcock et al. 2010). It should also be noted that the expression of *mfrA* was found to increase in the wildtype strain after exposure to H_2_O_2_ (data not shown), indicting that the Mfr is more abundunt in the prescence of the oxidant. Therefore, the mutant's exposure to H_2_O_2_ will, in principle, result in a decrease in the availability of free periplasmic iron that would normally be generated from the oxidation of MfrB. Since *C. jejuni* can transport iron to the intracellular milieu rapidly, this might explain the overall decrease in total intracellular iron in the Δ*mfrA* strain after H_2_O_2_-exposure as compared to the iron levels observed in the wildtype (Fig. 4). Specifically, although, like in other bacteria, the iron levels in both the wildtype and the Δ*mfrA* strain are expected to drop after exposure to an oxidant (Yamamoto et al. 2004), it is likely that absence of the Mfr will result in a further decrease in the intracellular iron levels of the mutant. Since iron is tightly regulated in the cell (Kakhlon and Cabantchik 2002; Kruszewski 2003), small changes in the concentration of intracellular iron might strongly impact the Fenton reaction as well as other factors (such as KatA) involved in H_2_O_2_ detoxification (van Vliet et al. 1999; Palyada et al. 2009; Kim et al. 2011). However, these observations require further investigation, because the generation of hydroxyl radicals, the byproduct of the interaction of H_2_O_2_ with iron in the Fenton reaction was only inferred from direct measurements of H_2_O_2_ accumulation in the Δ*mfrA* strain (Fig. 3).

Notably, mutants of other respiratory proteins with Fe-S centers, including hydrogenase (Hyd), nitrite reductase (Nrf), nitrate reductase (Nap), and formate dehydrogenase (Fdh) did not display H_2_O_2_ susceptibility phenotypes similar to that of the Δ*mfrA* strain (Kassem et al. 2012). Subsequently, we investigated the Mfr complex for unique features that might distinguish it from other respiratory proteins. We found that the Fe-S centers in the MfrB subunit have an unusual composition, being arranged as [2Fe-2S]-[4Fe-4S]-[4Fe-4S] (a binuclear and two tetranuclear centers) as compared to [2Fe-2S]-[3Fe-3S]-[4Fe-4S] that are typically found in succinate:quinone oxidoreductases and quinol:fumarate oxidoreductases (Lemos et al. 2002; Guccione et al. 2010), which obviously indicated that the Mfr has comparatively more iron stored in its Fe-S centers. However, the importance of these observations in terms of the interaction of the Mfr and H_2_O_2_ are currently not clear. Collectively, we propose that iron from Mfr might be working simultaneously with that released from other oxidation targets, including possibly other respiratory proteins, to maintain a delicate intracellular iron homeostasis that, in turn, influences the bacterium's response to H_2_O_2_.

Pathogens are challenged with an oxidative/respiratory burst, which comprises ROS, including H_2_O_2_, as a part of the macrophages’ first line of defense (Rosenberger and Finlay 2002; Imlay 2006; Slauch 2011). In fact, bacteria entrapped in the phagosomes encounter concentrations of H_2_O_2_ that may approach 10^−4^ mol/L (Imlay 2006). Therefore, deficiencies in *C. jejuni*'s tolerance to H_2_O_2_ normally coincide with the bacterium's decreased capability to persist in macrophages (Day et al. 2000; Atack and Kelly 2009). Subsequently, the increased persistence of the *ΔmfrA* strain in macrophages (Fig. 6A) can be explained, at least partially, by its decreased susceptibility to H_2_O_2_. Additionally, nitric oxide (NO) is produced by stimulated macrophages and also constitutes a component of the respiratory burst (Woodmansee and Imlay 2003). NO is known to inhibit several key enzymes (e.g., metalloproteins and thiol groups in proteins), as well as impair Fe-S centers (Poole 2005), while enhancing the rate of bacterial killing by H_2_O_2_ (Woodmansee and Imlay 2003). Therefore, the relatively elevated production of NO in the monolayers challenged with the *ΔmfrA* strain (Fig. 6C) may result from the increased persistence of the mutant in the co-cultures. Furthermore, the *ΔmfrA* strain resulted in morphological changes to the macrophage cell-shape, which persisted longer than those associated with the wildtype (Fig. 6B). It follows that the SEM analysis supported the capacity of the mutant to survive in higher numbers in macrophages as compared to the wildtype and corroborated previous findings that describe morphological changes to macrophages challenged with other pathogens (*Burkholderia pseudomallei* and *Pseudomonas aeruginosa*), toxins, or necrotic bodies (Kespichayawattana et al. 2000; Rocha et al. 2003).

We conclude that the Mfr is a potential indirect contributor to *C. jejuni*'s H_2_O_2_ response, likely via affecting intracellular iron concentrations and cognate H_2_O_2_ accumulation. This highlights the functional range of respiratory enzymes and the mechanisms that facilitate the survival of *C. jejuni*, a notably resilient microorganism.

## Supporting Information

Figure S1 Susceptibility of the Δ*mfrA* strain to organic peroxides (Cumene hydroperoxide and *tert*-butylhydroperoxide) and paraquat. Zones of inhibition were measured after incubation for 48 h. Microaerobic and anaerobic incubation conditions are abbreviated as “Micro” and “Ana,” respectively. Resistance to paraquat under anaerobic conditions could not be assessed under our experimental settings because of the interaction of the chemical with the pouches that generate anaerobic conditions. Statistically significant (*P* < 0.05) differences are highlighted with “*”. All experiments were repeated three times independently and samples were tested in at least three replicates per experiment. Data are presented as mean ± standard deviation.
