# Supplementary material for: The impairment of methylmenaquinol:fumarate reductase affects hydrogen peroxide susceptibility and accumulation in Campylobacter jejuni
Source: Microbiologyopen. 2014 Feb 7;3(2):168–81. doi: 10.1002/mbo3.158 (PMC3996566; doi:10.1002/mbo3.158)
Supplement: Figure S1 — Susceptibility of the ΔmfrA strain to organic peroxides (Cumene hydroperoxide and tert-butylhydroperoxide) and paraquat. Zones of inhibition were measured after incubation for 48 h. Microaerobic and anaerobic incubation conditions are abbreviated as “Micro” and “Ana,” respectively. Resistance to paraquat under anaerobic conditions could not be assessed under our experimental settings because of the interaction of the chemical with the pouches that generate anaerobic conditions. Statistically significant (P < 0.05) differences are highlighted with “*”. All experiments were repeated three times independently and samples were tested in at least three replicates per experiment. Data are presented as mean ± standard deviation. [file mbo30003-0168-sd1.pptx]

## Slide 1
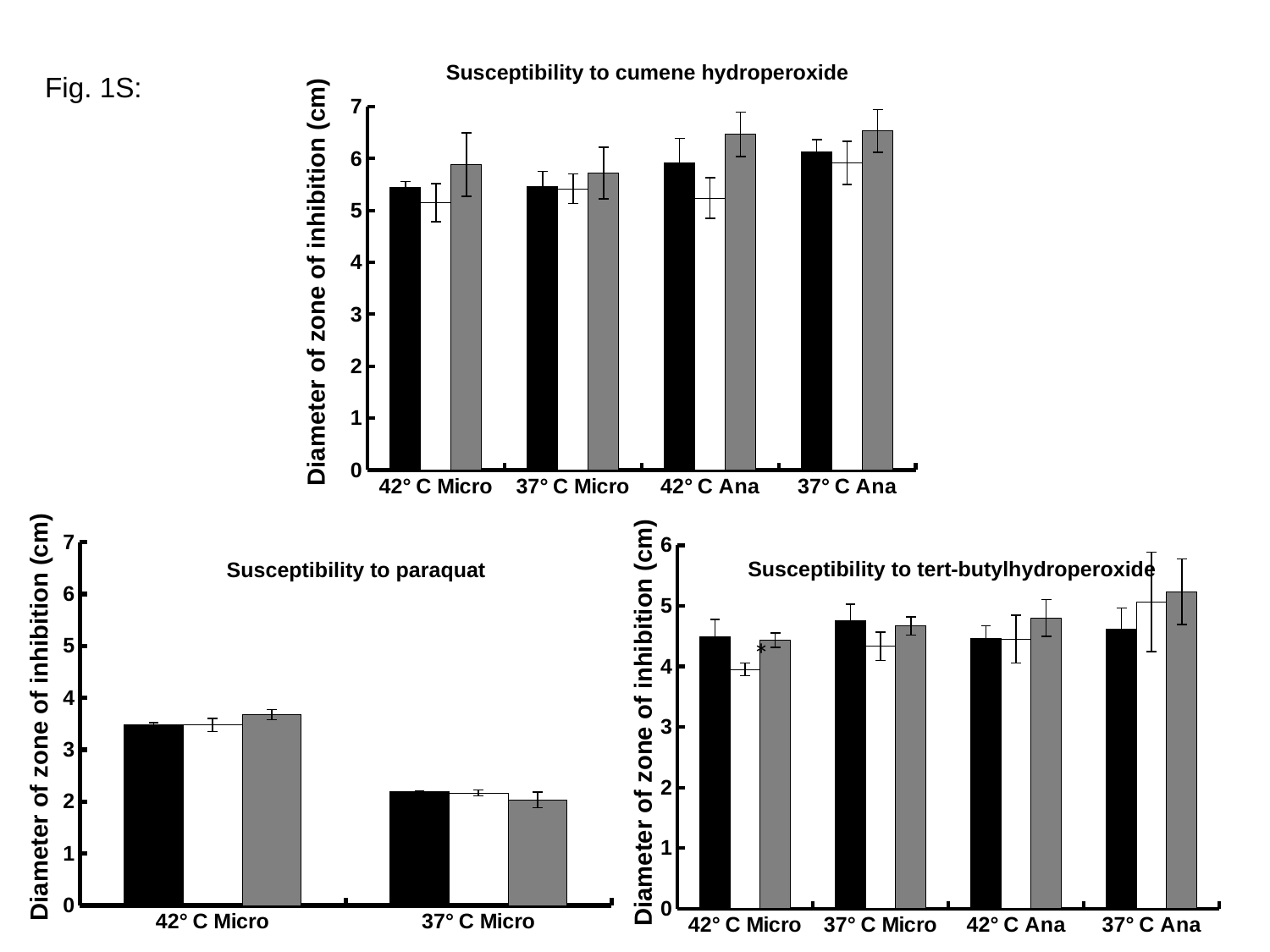

Susceptibility to cumene hydroperoxide
Fig. 1S:
### Chart
| Category | WT-11168 | ∆mfrA | mfrA-C |
|---|---|---|---|
| 42° C Micro | 5.449999999999999 | 5.1499999999999995 | 5.883333333333352 |
| 37° C Micro | 5.4666666666666694 | 5.416666666666671 | 5.72 |
| 42° C Ana | 5.916666666666671 | 5.24 | 6.4666666666666694 |
| 37° C Ana | 6.133333333333352 | 5.916666666666671 | 6.533333333333371 |Diameter of zone of inhibition (cm)
### Chart
| Category | WT-11168 | ∆mfrA | C-mfrA |
|---|---|---|---|
| 42° C Micro | 3.4749999999999988 | 3.4749999999999988 | 3.6750000000000003 |
| 37° C Micro | 2.2 | 2.1666666666666665 | 2.033333333333333 |Susceptibility to paraquat
### Chart
| Category | WT-11168 | ∆mfrA | mfrA-C |
|---|---|---|---|
| 42° C Micro | 4.48333333333338 | 3.9499999999999997 | 4.433333333333386 |
| 37° C Micro | 4.75 | 4.333333333333366 | 4.666666666666667 |
| 42° C Ana | 4.4666666666666694 | 4.45 | 4.8 |
| 37° C Ana | 4.616666666666666 | 5.066666666666666 | 5.23333333333338 |Susceptibility to tert-butylhydroperoxide
Diameter of zone of inhibition (cm)
Diameter of zone of inhibition (cm)
*

## Slide 2
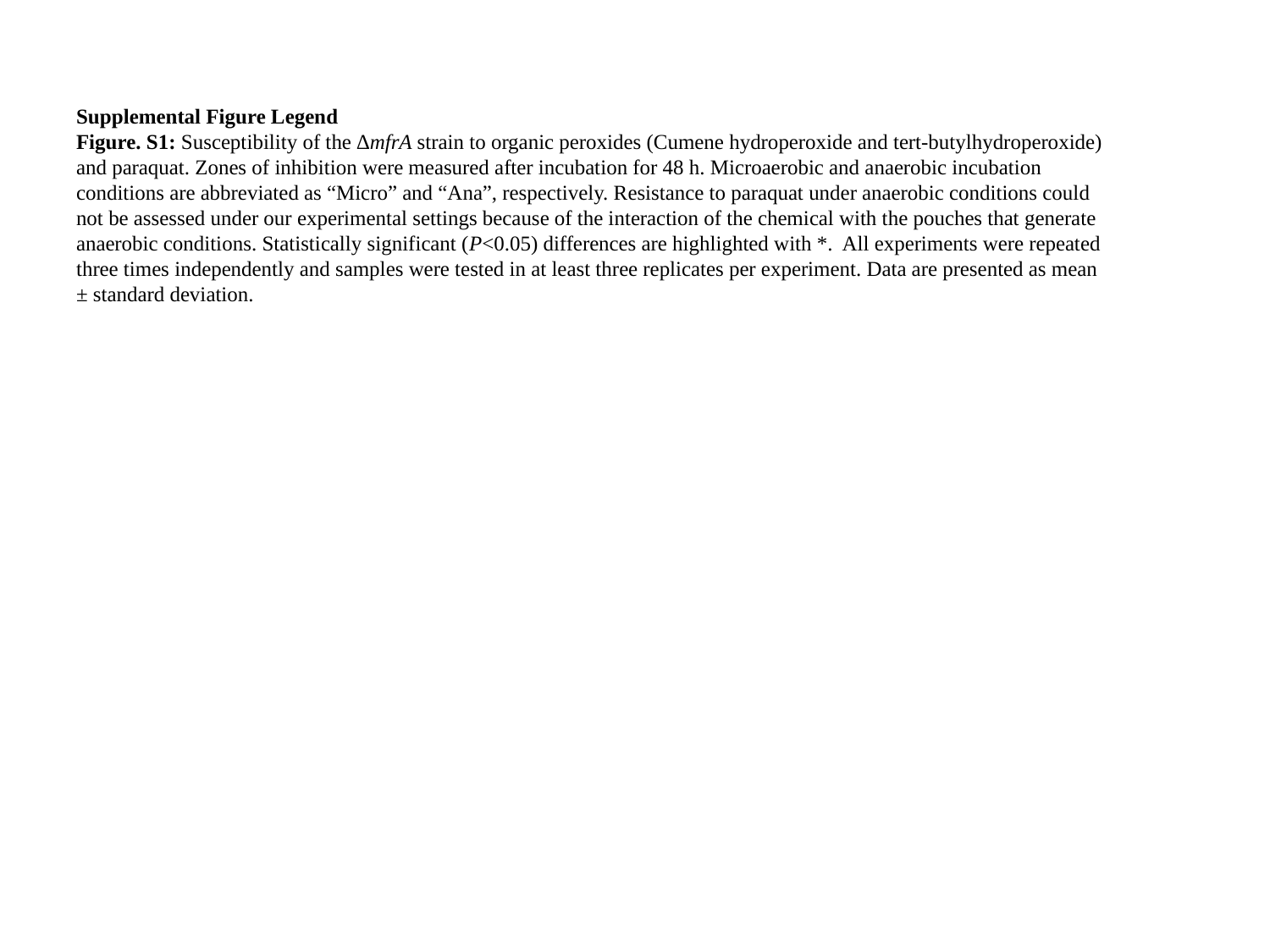

Supplemental Figure Legend
Figure. S1: Susceptibility of the ∆mfrA strain to organic peroxides (Cumene hydroperoxide and tert-butylhydroperoxide) and paraquat. Zones of inhibition were measured after incubation for 48 h. Microaerobic and anaerobic incubation conditions are abbreviated as “Micro” and “Ana”, respectively. Resistance to paraquat under anaerobic conditions could not be assessed under our experimental settings because of the interaction of the chemical with the pouches that generate anaerobic conditions. Statistically significant (P<0.05) differences are highlighted with *. All experiments were repeated three times independently and samples were tested in at least three replicates per experiment. Data are presented as mean ± standard deviation.
